# Toward a Functional Genetics of Adaptation: Insights From Microbial Experimental Evolution

**DOI:** 10.1093/gbe/evag158

**Published:** 2026-07-01

**Authors:** Rees Kassen

**Affiliations:** Department of Biology, McGill University, Montreal, Canada

**Keywords:** adaptation, genetics, microbial evolution, adaptive laboratory evolution, stress, gene regulation

## Abstract

A general theory of adaptation is one that accounts for both the quantitative and functional properties of adaptive evolution. To date, the field has focused more on the former and less on the latter. Here, I build on the results of adaptive laboratory evolution experiments in microbes to present a sketch for a functional theory of adaptation built around the idea that the primary source of selection in the initial stages of adaptation involves rapid re-growth in the presence of stress. Consequently, the genes targeted during adaptation are often those involved in the regulation of gene expression, including global regulators associated with managing the stress response. These mutations, especially those involving high level gene regulation, are often highly pleiotropic, in contrast to the expectation derived from the quantitative theory of adaptation. Whether this theory can be used to make sense of adaptation more generally beyond microbial evolution experiments remains an open issue.

Significance Evolutionary biology has developed a robust quantitative theory of adaptation that allows us to make predictions about the rate and extent of fitness increases. A comparable functional theory, one that can tell us about which genes and genetic changes are responsible for adaptation, has proven harder to articulate. This paper proposes a functional theory that sees the first steps of adaptation involving changes to gene regulation in response to stress. This explanation accords well with the results of laboratory experiments in microbial systems where many of the genetic changes associated with adaptation involve changes to global gene regulators and genes involved in controlling the expression of enzyme systems needed for growth. Whether this theory applies equally well to other systems remains to be seen.

## Introduction

Models of adaptation treat mutations as moves in phenotype (Fisher 1922; Orr 2005) or DNA sequence (Gillespie 1984; Orr 2002) space that result in a fitness increase. These models are, in essence, statistical descriptions of how rare events—beneficial mutations—behave under selection when a starting population is displaced from a fitness optimum. They have proven remarkably successful at predicting the number and effect size of mutations contributing to adaptation but, aside from the decision about whether to model phenotypes or DNA sequences, the theory contains rather little biology. Consequently, we know little about the functional genetic changes involved in adaptation and thus remain some way off from a general theory of the genetics of adaptation.

What might a functional theory of adaptation look like? At minimum it would predict the traits and genes involved in adaptation to novel conditions. While this is sometimes possible in special circumstances where selective targets are clear, such as antibiotic resistance in bacteria, more often we lack the prior knowledge to identify the phenotypic and genetic targets of selection. Instead, we track conspicuous and easily measured traits alongside a list of genetic changes whose connection to those traits is often tenuous because the functional effects of mutations are poorly characterized, unknown, or modulated by epistasis in ways hard to anticipate.

Making sense of these results is no easy task. One scenario where progress seems possible are adaptive laboratory evolution (ALE) experiments in microbes where populations are propagated in the laboratory and whole-genome sequencing is used to identify fixed or segregating mutations (Dettman et al. 2012; Kassen 2024). Sufficient numbers of these experiments are now available to begin making inferences about the nature of the functional changes underlying the first steps of adaptation in at least one context: a seasonal environment, alternating between periods of resource abundance and scarcity, that is stressful, in the sense that the founding lineage is mal-adapted to prevailing conditions. To the extent that these conditions capture features of the environment characterizing adaptation in other systems, microbial or otherwise, then they can be used to make sense of how functional adaptation might occur more generally.

## Phenotypic Changes in Growth Curves During Serial Transfer

ALE experiments typically propagate microbial populations via serial transfer in batch culture. A small volume of cells is removed from a dense culture and transferred into fresh media where it is allowed to expand before the process is repeated, usually every 24 to 48 h. The dynamics of microbial population growth in a single cycle are typically S-shaped and divided into three phases: acclimation or lag phase where cell division is slow as individuals adjust physiologically to the medium, exponential phase where division rates are high because resources are abundant, and stationary phase where division rates slow again as resources become limiting. Any number of models can capture these dynamics; the simplest and most common is the two-parameter logistic model, sometimes modified to include a third parameter capturing the lag phase (Ghenu et al. 2024). Growth curves are easy to measure and so are often used as proxies for fitness, making them a useful place to start thinking about the first steps of adaptation (Kassen 2024).

**Fig. 1.**
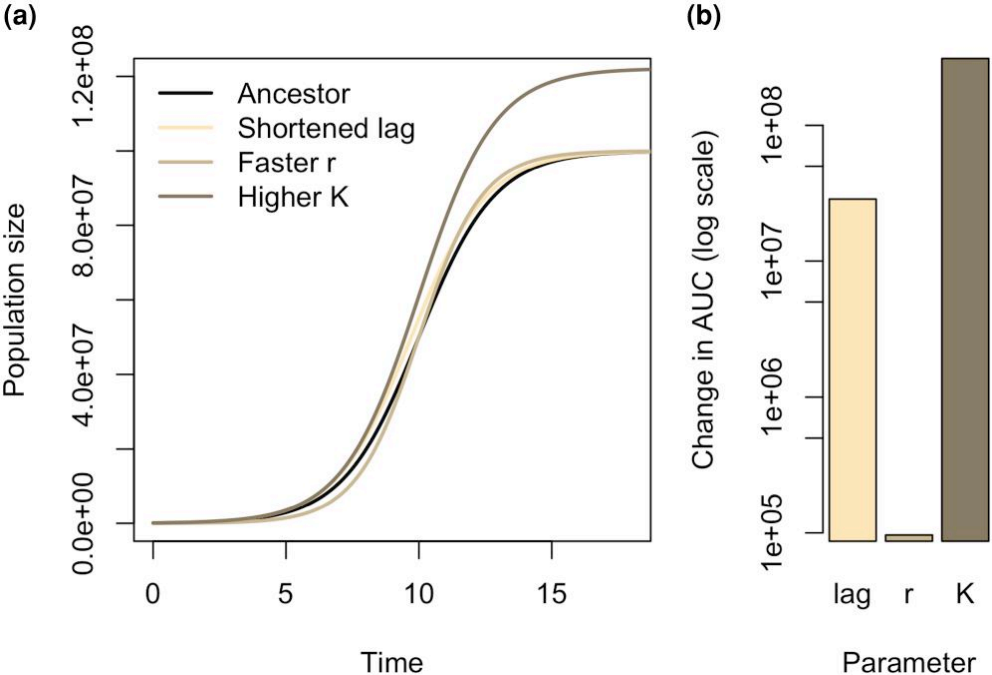
Fitness improvements can be governed by changes to components of the growth curve. a) Depicted are growth curves giving a 20% increase in fitness (selection coefficient, *s* = 0.2) over the ancestor (black line) corresponding to a reduction in lag phase (yellow) and increases to exponential growth rate (*r*; light brown) and stationary phase density (yield; dark brown). b) The same change in fitness (*s* = 0.2) leads to strikingly different increases in area under the curve (AUC) relative to the ancestor, the largest change being to yield and the smallest to *r*. The implication is that AUCs on their own provide little insight into which components of the growth curve are responding to selection. Further details on calculations provided in File S1.

Adaptation is the result of increases in the rate of replication of one lineage over another. In the absence of non-transitive interactions among lineages (Houpt and Kassen 2023) or frequency-dependent selection (Rainey and Travisano 1998), increases in replication rates are usually realized through changes in the components of the growth curve (Fig. 1). Fitness can increase because, relative to the ancestor, the duration of lag phase decreases, exponential growth rate increases, or stationary phase density is higher. In practice, most studies report exponential growth rates or stationary phase density (Ghenu et al. 2024), likely because lag phase requires measuring cell densities below the detection limit of most automated plate readers. Consequently, only a handful of experiments have examined how all three components of growth change due to selection in batch culture.

Of the experiments that have estimated all three components of growth, we see a striking result: all show a compression of the lag phase following selection (Vasi et al. 1994; Oxman et al. 2008; Li et al. 2018; Moreno-Gámez et al. 2020), consistent with the idea that rapid re-growth upon transfer to fresh media is a trait under selection. Other components of the growth curve also change, although less consistently: rates of exponential growth can increase, in one case after reductions in what was initially a long lag phase and in another when lag phase was initially greatly reduced due to the addition of a preferred carbon source (Oxman et al. 2008) and stationary phase density either increases or decreases. Decreases in stationary phase density are often tied to increases in cell size (Vasi et al. 1994; Li et al. 2018) perhaps reflecting the value of having larger cells carry over more resources to re-start growth in the next growth cycle.

**Fig. 2.**
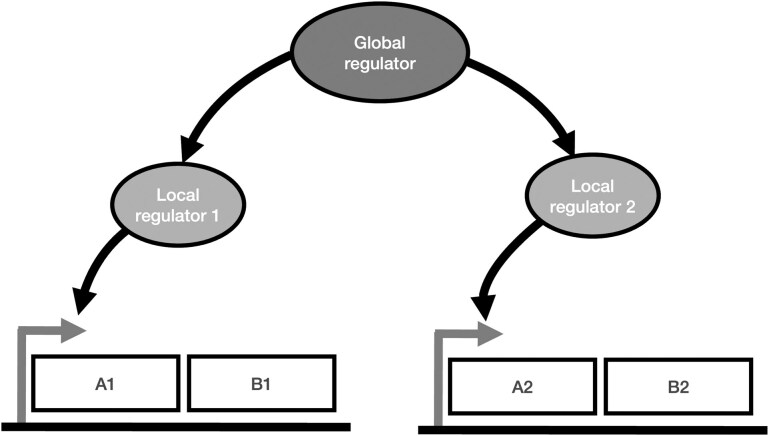
The modular hierarchical view of gene expression. Expression of genes A and B within each of operons 1 and 2 are under the control of local promoters (gray arrows) that are controlled by separate local regulators 1 and 2, respectively, which in turn are controlled by a global regulator.

What genetic changes are responsible for the rapid re-growth associated with reductions in lag phase? Two studies point to modified gene regulation as the cause. Selection for rapid re-growth of *Escherichia coli* on lactose after starvation reduced lag times from approximately 10 h down to 4 h caused by loss of function mutations in *lacI*, a transcriptional repressor, leading to constitutive expression of the lac operon, (Moreno-Gámez et al. 2020). The first beneficial mutations in yeast adapting to glucose also reduced lag time likely because increased respiration, though not fermentation, rates led to larger cells that rapidly regrow following transfer to fresh media due to upregulation of the Ras/protein kinase A nutrient stress response pathway (Li et al. 2018). These two results exemplify a recurring theme in ALE experiments: many of the genetic changes recovered from evolved populations impact gene regulation in some way (Dettman et al. 2012; Hindré et al. 2012; Huang et al. 2018; Wang et al. 2018; McDonald 2019; Johnson et al. 2021; Lennen et al. 2023; Kassen 2024; Martini et al. 2025). Below I present a conceptual and mechanistic framework for why this is the case (Fig. 2).

## Gene Regulation as a Target of Selection: The Modular Hierarchical Model

A conceptual framework for understanding the role of gene regulation in ALE experiments comes from the modular hierarchical nature of gene expression depicted in Fig. 2. The lowest level of the hierarchy consists of structural genes, those that code for protein products like enzymes (blocks denoted A1 and B1 in the figure), whose expression is under the control of upstream regulatory regions (the vertical line connected to the arrow representing the direction of transcription) to which transcription factors bind. The expression of the *lac* operon in *E. coli* is the canonical example: the repressor, *lacI*, prevents expression of the genes involved in transport (*lacY*), cleaving (*lacZ*), and modification (*lacA*) of lactose (Dekel and Alon 2005). The expression of these lower-level units is controlled by “local” regulatory genes such as, in the case of lac, the cAMP repressor protein that recruits RNA polymerase to the lac promoter in the absence of glucose (Phillips et al. 2019). Local regulation can in turn be controlled by “global” regulatory genes such as RNA polymerases (Battesti et al. 2011) or those controlling the supercoiling structure of DNA that modulate the ability of transcription factors to bind to DNA (Hatfield and Benham 2002).

The result is a modular network of hierarchically controlled regulation governing the expression of structural genes and the enzymes they produce. In this light, it is easy to see how changes to different levels of the hierarchy will cause changes in gene expression that impact, at the lowest level, modular units like the *lac* operon or, if mutations occur in global regulatory genes, a much wider suite of genes that causes highly pleiotropic effects across the entire gene expression network.

## Two Mechanistic Explanations Why Gene Regulation Is a Target of Selection

The modular hierarchical network model suggests a simple reason gene regulation can be a common target of selection: loss-of-function mutations (where “loss-of-function” refers to the reduction or elimination of the protein product of a gene, not the effect on fitness), which are common because there are often many more ways to disrupt function (nonsense mutations, indel-driven frameshifts, or large deletions) than to improve it, in regulatory genes, especially those serving as repressors, can lead to increased expression of downstream gene networks linked to replication rates. Two mechanisms involving gene regulation are likely contributors to rapid re-growth being a target of selection in microbial evolution experiments.

### Re-balancing the Stress–Growth Trade-off

Microbes possess a range of mechanisms allowing them to respond to changing environmental conditions. Among the most important are the general and stringent responses, that act as global regulators of gene expression in response to stressors such as nutrient depletion and osmotic or heat shock (Bergkessel et al. 2016). Rapid growth under nutrient replete conditions is supported by maximizing ribosome, and so protein, biosynthesis (Scott et al. 2010). Under stressful conditions, growth rate slows because the general and stringent responses slow transcription, reducing rates of gene expression and shunting ribosome production to genes responsible for dealing with the stress itself or managing DNA damage. The balance between growth and stress responses likely represents a fundamental trade-off to which all microbes must contend (Ferenci 2005; Ferenci and Spira 2007).

This trade-off likely plays a central role in ALE experiments. The conditions of growth in the laboratory are invariably stressful, if only because daily transfer in batch-culture leads to regular transitions between periods of nutrient abundance soon after transfer and nutrient limitation during stationary phase, once nutrients have been depleted. ALE experiments can also be stressful by design, since without a source of stress like antibiotics, pH, osmolarity, or heat there would be little opportunity for adaptation and not much to study. If the primary physiological state of cells during the initial phases of a selection experiment is stress, and responding to stress comes at the price of vegetative growth, then any mutation allowing growth to resume in the face of stress is likely to be favored by selection.

The evidence to support this argument is that selection on high level regulation is, as noted above, a common feature of microbial evolution experiments (Dettman et al. 2012; Hindré et al. 2012; Saxer et al. 2014; Wang et al. 2018; Chen and Zhang 2024). High level transcription controls adjusting RNA polymerase activity, achieved through cofactors of the general and stringent stress responses, such as *rpoS, hfq, spoT, rpoB*, and *rpoC*, are common targets in bacterial selection experiments (Wang et al. 2018; Choudhury et al. 2023), as are genes like *topA*, *fis*, and *dusB* implicated in DNA topology, and so the ability of promoters to bind to DNA and modulate expression (reviewed in Dettman et al. 2012; Zorraquino et al. 2016). In yeast, mutations in the the Spt-Ada-Gcn5-acetyltransferase-mediated stress response and other targets of gene expression such as signal transduction (*IRA2*), nutrient transport (*GNP1*), chromatin remodeling, transcription, translation, and cell growth are also recovered (Anderson et al. 2010; Araya et al. 2010; Kvitek and Sherlock 2013; Venkataram et al. 2016; Chen and Zhang 2024).

### De-regulation Leading to Constitutive Expression

A second explanation involves changes to gene expression due to de-regulation of an otherwise inducible system. When enzyme function limits growth, fitness can often increase either by making more enzyme or improving the activity of the enzymes already present. One of the easiest ways to make more enzyme is a loss-of-function mutation that knocks out the regulatory function of a key pathway and leads to constitutive expression of the native enzyme function. The de-regulating mutations in *lacI*, a transcriptional repressor of the *lac* operon (Moreno-Gámez et al. 2020), that led to the evolution of reduced lag phase in *E. coli* selected on lactose are an example of this mechanism.

De-regulation mutations are commonly observed in ALE experiments. These include adaptation to novel carbon sources in *Klebsiella* (Lin et al. 1976), quorum sensing, and metabolism via *lasR* in *Pseudomonas aeruginosa* (Mould et al. 2022; Schick et al. 2022), biofilm formation associated with occupation of the air–broth interface in *Pseudomonas fluorescens* (Bantinaki et al. 2007; McDonald et al. 2011), the emergence of “fast-switching” *E. coli* variants that shift rapidly from glucose to acetate when both resources are present in the media (Friesen et al. 2004; Spencer et al. 2007), and nutrient response associated in yeast populations adapting to limited glucose (Venkataram et al. 2016). No doubt many other examples exist.

## First-step Mutations Are Often Highly Pleiotropic

If the first steps of adaptation occur through global regulatory mutations, they are likely to be highly pleiotropic. This prediction seems to stand in contrast to that of Fisher's geometric model of adaptation, where highly pleiotropic beneficial mutations are less likely to be fixed close to the fitness optimum because it is harder for a single mutation to improve multiple traits simultaneously than it is to improve just one (Orr 2000). Which of these views is more accurate?

Work by Kinsler et al. (2024) suggests the first mutations fixed are more pleiotropic than later ones. In glucose-limited environments, the growth phases of yeast include a lag phase involving physiological acclimation followed by fermentation of glucose to ethanol, respiration of ethanol, and then a quiescent stationary phase. Far more first-step mutations improved both respiration and fermentation (85%) than second-step mutations (35%), with the majority of the latter resulting in improved respiration but not fermentation. The identity of mutations in the first and second step also differed: first-step mutations occurred in one of two signaling pathways (Ras/PKA or TOR/Sch9) that led to constitutive expression of both whereas second-step mutations showed a signal of enriching for mutations in more specific pathways involving regulation of respiration and mitochondrial function.

It is too early to tell whether such a progression from high to low pleiotropy over the course of an adaptive walk is a general one. It is notable, though, that a meta-analysis of genes mutated between 500 and 2,000 generations of selection across a wide range of evolution experiments in *E. coli* revealed that the connectivity (meaning protein–protein connections, a proxy for pleiotropy) of mutated genes tended to be higher than those of non-mutated genes (Ruelens et al. 2023). Notably, among the most connected, and so presumably pleiotropic, genes mutated were those involved in gene expression, especially RNA polymerase genes like *rpoB* and *rpoC.* Global regulators of gene expression are, therefore, both more likely to contribute to adaptation—likely due to their ability to facilitate rapid re-growth in response to stress—and be highly pleiotropic.

## Discussion

A general theory of adaptation is one that allows us to make predictions about the rate and magnitude of fitness change under selection and the functional properties of the genes involved. Much progress has been made on the quantitative side by framing adaptation as a statistical problem, where mutations are treated as draws from an underlying distribution of fitness effects (Orr 2005; Good et al. 2012; Buskirk et al. 2017; Kassen 2024). Far less progress has been made on the functional side.

My contention is that many of the mutations contributing to the first steps of adaptation of are those that permit rapid re-growth in response to stress. A phenotypic signal of this response is seen in batch culture ALE experiments where reductions in the duration of lag phase are a common evolved response to serial transfer. This is not an original observation. Microbiologists, as part of the regular practice of acclimating (or, more accurately, domesticating) wild strains to the lab, will have no doubt noticed similar changes to the growth properties of their favorite strain. Nevertheless, microbiologists have remained attached to exponential growth, and to some extent stationary phase density, as the traits that matter most for fitness. As a result, it remains rare for all components of the growth curve—lag phase, exponential growth rates, and stationary phase density—to be measured in an evolutionary meaningful way. In the handful of studies where this has been done, all components of the growth curve can change, including sometimes striking decreases in the duration of lag phase, following selection. Reduced lag phase is consistent with the idea that rapid re-growth is a target of selection.

Closer examination of the genetic changes associated with reduced lag phase and other early arising mutations suggest altered gene regulation as a cause. The hierarchical modular view of gene expression and regulation provides a useful framework for understanding why rapid re-growth results from modifications that allow growth to proceed in the presence of stress or from de-regulation of inducible enzyme systems to make them constitutive, relieving enzyme-limiting metabolic processes. Both mechanisms often stem from loss-of-function mutations, which helps explain why they are commonly observed in the early stages of adaptation. Moreover, this framework can explain why many early-stage beneficial mutations are more pleiotropic than later ones: global gene regulatory changes that re-balance the stress-growth trade-off are likely to be highly pleiotropic, as they impact many lower-level gene expression networks. More work could be done to test this hypothesis.

Regulatory changes are not the only genetic route to adaptation observed in microbial evolution experiments. Adaptation to laboratory conditions, a form of domestication, through loss of function mutations or deletions impacting traits such as motility (Kearns and Losick 2003; Maughan and Nicholson 2011; Bailey et al. 2015; Schick and Kassen 2018; Barreto et al. 2020; Sher et al. 2020), virulence factor production (Somerville et al. 2002; Barreto et al. 2020), and cell-to-cell connections allowing aggregation and biofilm formation (Surve et al. 2024; Gifford et al. 2025) are often observed. Increased gene dosage, often via whole-genome duplications or smaller-scale gene amplifications, are common because they increase the quantity of enzymes or processes limiting to growth (Gerstein et al. 2006; Bergthorsson et al. 2007; Gresham et al. 2008; Venkataram et al. 2016; Li et al. 2018), similar to the effect of de-regulation mutations. How domestication and gene dosage changes impact pleiotropy during adaptation deserves closer attention.

Whether this framework constitutes a general explanation for the first steps of adaptation under culture conditions outside the lab remains an open issue. In microbial systems, at least, there is some indication that changes to gene regulation occur readily in more natural settings. Adaptation by *E. coli* to the mouse gut repeatedly involved inactivation mutations of regulator genes including *lacI* and *dgoR* amongst other metabolic genes, in addition to the loss of motility (Lescat et al. 2017; Ghalayini et al. 2019; Vasquez et al. 2021). We and others commonly recover mutations in the global regulator *lasR* across a wide range of conditions in *Pseudomonas aeruginosa* strains associated with chronic infection of the cystic fibrosis respiratory tract (Hoffman et al. 2009; Mould et al. 2022; Schick et al. 2022). Such repeated evolution suggests changes in gene regulation associated with managing stress could be a general route to adaptation. At the very least, this explanation provides an empirically testable hypothesis we can use to start building out a more general theory of adaptation that accounts for both the functional and statistical properties of beneficial mutations.

## Supplementary Material

evag158_Supplementary_Data

## Data Availability

No new data were generated or analyzed in support of this research.

## References

[evag158-B1] Anderson JB, et al Determinants of divergent adaptation and Dobzhansky-Muller interaction in experimental yeast populations. Curr Biol. 2010:20:1383–1388. 10.1016/j.cub.2010.06.022. 20637622 PMC2938792

[evag158-B2] Araya CL, Payen C, Dunham MJ, Fields S. Whole-genome sequencing of a laboratory-evolved yeast strain. BMC Genomics. 2010:11:88. 10.1186/1471-2164-11-88. 20128923 PMC2829512

[evag158-B3] Bailey SF, Rodrigue N, Kassen R. The effect of selection environment on the probability of parallel evolution. Mol Biol Evol. 2015:32:1436–1448. 10.1093/molbev/msv033. 25761765

[evag158-B4] Bantinaki E, et al Adaptive divergence in experimental populations of *Pseudomonas fluorescens*. III. Mutational origins of wrinkly spreader diversity. Genetics. 2007:176:441–453. 10.1534/genetics.106.069906. 17339222 PMC1893022

[evag158-B5] Barreto HC, Cordeiro TN, Henriques AO, Gordo I. Rampant loss of social traits during domestication of a Bacillus subtilis natural isolate. Sci Rep. 2020:10:18886. 10.1038/s41598-020-76017-1. 33144634 PMC7642357

[evag158-B6] Battesti A, Majdalani N, Gottesman S. The RpoS-mediated general stress response in *Escherichia coli*. Annu Rev Microbiol. 2011:65:189–213. 10.1146/annurev-micro-090110-102946. 21639793 PMC7356644

[evag158-B7] Bergkessel M, Basta DW, Newman DK. The physiology of growth arrest: uniting molecular and environmental microbiology. Nat Rev Microbiol. 2016:14:549–562. 10.1038/nrmicro.2016.107. 27510862 PMC10069271

[evag158-B8] Bergthorsson U, Andersson DI, Roth JR. Ohno’s dilemma: evolution of new genes under continuous selection. Proc Natl Acad Sci U S A. 2007:104:17004–17009. 10.1073/pnas.0707158104. 17942681 PMC2040452

[evag158-B9] Buskirk S, Peace RE, Lang GI. Hitchhiking and epistasis give rise to cohort dynamics in adapting populations. Proc Natl Acad Sci U S A. 2017:114:8330–8335. 10.1073/pnas.1702314114. 28720700 PMC5547604

[evag158-B10] Chen P, Zhang J. The loci of environmental adaptation in a model eukaryote. Nat Commun. 2024:15:5672. 10.1038/s41467-024-50002-y. 38971805 PMC11227561

[evag158-B11] Choudhury A, et al Deep mutationalscanning reveals the molecular determinants of RNA polymerase-mediated adaptation and tradeoffs. Nat Commun. 2023:14:6319. 10.1038/s41467-023-41882-7. 37813857 PMC10562459

[evag158-B12] Dekel E, Alon U. Optimality and evolutionary tuning of the expression level of a protein. Nature. 2005:436:588–592. 10.1038/nature03842. 16049495

[evag158-B13] Dettman JR, et al Evolutionary insight from whole-genome sequencing of experimentally evolved microbes. Mol Ecol. 2012:21:2058–2077. 10.1111/j.1365-294X.2012.05484.x. 22332770

[evag158-B14] Ferenci T . Maintaining a healthy SPANC balance through regulatory and mutational adaptation: stress resistance and metabolic capability. Mol Microbiol. 2005:57:1–8. 10.1111/j.1365-2958.2005.04649.x. 15948944

[evag158-B15] Ferenci T, Spira B. Variation in stress responses within a bacterial species and the indirect costs of stress resistance. Ann N Y Acad Sci. 2007:1113:105–113. 10.1196/annals.1391.003. 17483210

[evag158-B16] Fisher RA . Darwinian evolution of mutations. Eugen Rev. 1922:14:31–34. 21259738 PMC2942494

[evag158-B17] Friesen ML, Saxer G, Travisano M, Doebeli M. Experimental evidence for sympatric ecological diversification due to frequency-dependent competition in *Escherichia coli*. Evolution. 2004:58:245–260. 10.1111/j.0014-3820.2004.tb01642.x. 15068343

[evag158-B18] Gerstein AC, Chun H-JE, Grant A, Otto SP. Genomic convergence toward diploidy in *Saccharomyces cerevisiae*. PLoS Genet. 2006:2:e145. 10.1371/journal.pgen.0020145. 17002497 PMC1570378

[evag158-B19] Ghalayini M, et al Long-term evolution of the natural isolate of *Escherichia coli* 536 in the mouse gut colonized after maternal transmission reveals convergence in the constitutive expression of the lactose operon. Mol Ecol. 2019:28:4470–4485. 10.1111/mec.15232. 31482587

[evag158-B20] Ghenu A-H, Marrec L, Bank C. Challenges and pitfalls of inferring microbial growth rates from lab cultures. Front Ecol Evol. 2024:11:1313500. 10.3389/fevo.2023.1313500.

[evag158-B21] Gifford I, Vergis MR, Barrick JE. Genome evolution of *Acinetobacter baylyi* ADP1 during laboratory domestication: acquired mutations impact competence and metabolism. Appl Environ Microbiol. 2025:91:e00936-25. 10.1128/aem.00936-25. 40827875 PMC12442408

[evag158-B22] Gillespie JH . Molecular evolution over the mutational landscape. Evolution. 1984:38:1116–1129. 10.2307/2408444. 28555784

[evag158-B23] Good BH, Rouzine IM, Balick DJ, Hallatschek O, Desai MM. Distribution of fixed beneficial mutations and the rate of adaptation in asexual populations. Proc Natl Acad Sci U S A. 2012:109:4950–4955. 10.1073/pnas.1119910109. 22371564 PMC3323973

[evag158-B24] Gresham D, et al The repertoire and dynamics of evolutionary adaptations to controlled nutrient-limited environments in yeast. PLoS Genet. 2008:4:e1000303. 10.1371/journal.pgen.1000303. 19079573 PMC2586090

[evag158-B25] Hatfield GW, Benham CJ. DNA topology-mediated control of global gene expression in *Escherichia coli*. Annu Rev Genet. 2002:36:175–203. 10.1146/annurev.genet.36.032902.111815. 12429691

[evag158-B26] Hindré T, Knibbe C, Beslon G, Schneider D. New insights into bacterial adaptation through in vivo and in silico experimental evolution. Nat Rev Microbiol. 2012:10:352–365. 10.1038/nrmicro2750. 22450379

[evag158-B27] Hoffman LR, et al *Pseudomonas aeruginosa lasR* mutants are associated with cystic fibrosis lung disease progression. J Cyst Fibros. 2009:8:66–70. 10.1016/j.jcf.2008.09.006. 18974024 PMC2631641

[evag158-B28] Houpt NSB, Kassen R. On the *de novo* emergence of ecological interactions during evolutionary diversification: a conceptual framework and experimental test. Am Nat. 2023:202:800–817. 10.1086/726895. 38033179

[evag158-B29] Huang C-J, Lu M-Y, Chang Y-W, Li W-H. Experimental evolution of yeast for high-temperature tolerance. Mol Biol Evol. 2018:35:1823–1839. 10.1093/molbev/msy077. 29684163

[evag158-B30] Johnson MS, et al Phenotypic and molecular evolution across 10,000 generations in laboratory budding yeast populations. eLife. 2021:10:e63910. 10.7554/eLife.63910. 33464204 PMC7815316

[evag158-B31] Kassen R . Experimental evolution and the nature of biodiversity. 2nd ed. Oxford University Press; 2024.

[evag158-B32] Kearns DB, Losick R. Swarming motility in undomesticated *Bacillus subtilis*. Mol Microbiol. 2003:49:581–590. 10.1046/j.1365-2958.2003.03584.x. 12864845

[evag158-B33] Kinsler G, Li Y, Sherlock G, Petrov DA. A high-resolution two-step evolution experiment in yeast reveals a shift from pleiotropic to modular adaptation. PLoS Biol. 2024:22:e3002848. 10.1371/journal.pbio.3002848. 39636818 PMC11620474

[evag158-B34] Kvitek DJ, Sherlock G. Whole genome, whole population sequencing reveals that loss of signaling networks is the major adaptive strategy in a constant environment. PLOS Genet. 2013:9:e1003972. 10.1371/journal.pgen.1003972. 24278038 PMC3836717

[evag158-B35] Lennen RM, et al Laboratory evolution reveals general and specific tolerance mechanisms for commodity chemicals. Metab Eng. 2023:76:179–192. 10.1016/j.ymben.2023.01.012. 36738854

[evag158-B36] Lescat M, et al Using long-term experimental evolution to uncover the patterns and determinants of molecular evolution of an *Escherichia coli* natural isolate in the streptomycin-treated mouse gut. Mol Ecol. 2017:26:1802–1817. 10.1111/mec.13851. 27661780 PMC5734618

[evag158-B37] Li Y, et al Hidden complexity of yeast adaptation under simple evolutionary conditions. Curr Biol. 2018:28:515–525.e6. 10.1016/j.cub.2018.01.009. 29429618 PMC5823527

[evag158-B38] Lin ECC, Hacking AJ, Aguilar J. Experimental models of acquisitive evolution. BioScience. 1976:26:548–555. 10.2307/1297270.

[evag158-B39] Martini AM, Alexander SA, Khare A. Mutations in the *Staphylococcus aureus* global regulator *CodY* confer tolerance to an interspecies redox-active antimicrobial. PLoS Genet. 2025:21:e1011610. 10.1371/journal.pgen.1011610. 40053555 PMC11918324

[evag158-B40] Maughan H, Nicholson WL. Increased fitness and alteration of metabolic pathways during *Bacillus subtilis* evolution in the laboratory. Appl Environ Microbiol. 2011:77:4105–4118. 10.1128/AEM.00374-11. 21531833 PMC3131648

[evag158-B41] McDonald MJ . Microbial experimental evolution – a proving ground for evolutionary theory and a tool for discovery. EMBO Rep. 2019:20:e46992. 10.15252/embr.201846992. 31338963 PMC6680118

[evag158-B42] McDonald MJ, Cooper TF, Beaumont HJE, Rainey PB. The distribution of fitness effects of new beneficial mutations in *Pseudomonas fluorescens*. Biol Lett. 2011:7:98–100. 10.1098/rsbl.2010.0547. 20659918 PMC3030884

[evag158-B43] Moreno-Gámez S, et al Wide lag time distributions break a trade-off between reproduction and survival in bacteria. Proc Natl Acad Sci U S A. 2020:117:18729–18736. 10.1073/pnas.2003331117. 32669426 PMC7414188

[evag158-B44] Mould DL, Stevanovic M, Ashare A, Schultz D, Hogan DA. Metabolic basis for the evolution of a common pathogenic *Pseudomonas aeruginosa* variant. eLife. 2022:11:e76555. 10.7554/eLife.76555. 35502894 PMC9224983

[evag158-B45] Orr HA . Adaptation and the cost of complexity. Evolution. 2000:54:13–20. 10.1111/j.0014-3820.2000.tb00002.x. 10937178

[evag158-B46] Orr HA . The population genetics of adaptation: the adaptation of DNA sequences. Evolution. 2002:56:1317–1330. 10.1111/j.0014-3820.2002.tb01446.x. 12206234

[evag158-B47] Orr HA . The genetic theory of adaptation: a brief history. Nat Rev Genet. 2005:6:119–127. 10.1038/nrg1523. 15716908

[evag158-B48] Oxman E, Alon U, Dekel E. Defined order of evolutionary adaptations: experimental evidence. Evolution. 2008:62:1547–1554. 10.1111/j.1558-5646.2008.00397.x. 18410537

[evag158-B49] Phillips KN, et al Diversity in *lac* operon regulation among diverse *Escherichia coli* isolates depends on the broader genetic background but is not explained by genetic relatedness. mBio. 2019:10:e02232-19. 10.1128/mBio.02232-19. 31719176 PMC6851279

[evag158-B50] Rainey PB, Travisano M. Adaptive radiation in a heterogeneous environment. Nature. 1998:394:69. 10.1038/27900. 9665128

[evag158-B51] Ruelens P, Wynands T, De Visser JAGM. Interaction between mutation type and gene pleiotropy drives parallel evolution in the laboratory. Philosophical Transactions of the Royal Society B. 2023:378:20220051. 10.1098/rstb.2022.0051. PMC10067263 37004729

[evag158-B52] Saxer G, et al Mutations in global regulators lead to metabolic selection during adaptation to complex environments. PLoS Genet. 2014:10:e1004872. 10.1371/journal.pgen.1004872. 25501822 PMC4263409

[evag158-B53] Schick A, Kassen R. Rapid diversification of *Pseudomonas aeruginosa* in cystic fibrosis lung-like conditions. Proc Natl Acad Sci U S A. 2018:115:10714–10719. 10.1073/pnas.1721270115. 30275334 PMC6196507

[evag158-B54] Schick A, Shewaramani S, Kassen R. Genomics of diversification of *Pseudomonas aeruginosa* in cystic fibrosis lung-like conditions. Genome Biol Evol. 2022:14:evac074. 10.1093/gbe/evac074. 35660861 PMC9168666

[evag158-B55] Scott M, Gunderson CW, Mateescu EM, Zhang Z, Hwa T. Interdependence of cell growth and gene expression: origins and consequences. Science. 2010:330:1099–1102. 10.1126/science.1192588. 21097934

[evag158-B56] Sher AA, et al Experimental evolution of *Campylobacter jejuni* leads to loss of motility, *rpoN* (σ54) deletion and genome reduction. Front Microbiol. 2020:11:579989. 10.3389/fmicb.2020.579989. 33240235 PMC7677240

[evag158-B57] Somerville GA, et al *In vitro* serial passage of *Staphylococcus aureus*: changes in physiology, virulence factor production, and *agr* nucleotide sequence. J Bacteriol. 2002:184:1430–1437. 10.1128/JB.184.5.1430-1437.2002. 11844774 PMC134861

[evag158-B58] Spencer CC, Bertrand M, Travisano M, Doebeli M. Adaptive diversification in genes that regulate resource use in *Escherichia coli*. PLoS Genet. 2007:3:e15. 10.1371/journal.pgen.0030015. 17238290 PMC1779306

[evag158-B59] Surve SV, et al Laboratory domestication of *Lactiplantibacillus plantarum* alters some phenotypic traits but causes non-novel genomic impact. J Appl Microbiol. 2024:135:lxae035. 10.1093/jambio/lxae035. 38341274

[evag158-B60] Vasi F, Travisano M, Lenski RE. Long-term experimental evolution in *Escherichia coli*. II. Changes in life-history traits during adaptation to a seasonal environment. Am Nat. 1994:144:432–456. 10.1086/285685.

[evag158-B61] Vasquez KS, et al Quantifying rapid bacterial evolution and transmission within the mouse intestine. Cell Host Microbe. 2021:29:1454–1468.e4. 10.1016/j.chom.2021.08.003. 34473943 PMC8445907

[evag158-B62] Venkataram S, et al Development of a comprehensive genotype-to-fitness map of adaptation-driving mutations in yeast. Cell. 2016:166:1585–1596.e22. 10.1016/j.cell.2016.08.002. 27594428 PMC5070919

[evag158-B63] Wang X, Zorraquino V, Kim M, Tsoukalas A, Tagkopoulos I. Predicting the evolution of Escherichia coli by a data-driven approach. Nat Commun. 2018:9:3562. 10.1038/s41467-018-05807-z. 30177705 PMC6120903

[evag158-B64] Zorraquino V, Kim M, Rai N, Tagkopoulos I. The genetic and transcriptional basis of short and long term adaptation across multiple stresses in *Escherichia coli*. Mol Biol Evol. 2016:34:707–717. 10.1093/molbev/msw269. 28007978

