# Supplementary material for: Toward a Functional Genetics of Adaptation: Insights From Microbial Experimental Evolution
Source: Genome Biol Evol. 2026 Jul 1;18(7):evag158. doi: 10.1093/gbe/evag158 (PMC13335803; doi:10.1093/gbe/evag158)
Supplement: evag158_Supplementary_Data [file evag158_supplementary_data.pdf]

Supplementary file #1 - Growth curve components under selection

Kassen (2026) "Towards a functional genetics of adaptation: insights from microbial evolution experiments"

Logistic population growth with a lag phase

The standard logistic population growth equation can be modified to include an acclimation or lag phase as follows:

$$N(t) = \frac{K}{1 + e^{-rmax(t-lag)}}$$

Where *N(t)* is the population size at time *t*, *K* is the carrying capacity or stationary phase density, *rmax* is the maximum per capita growth rate (when resources are not limiting) and *lag* is the duration of the acclimation period before growth begins. The result is an S-shaped population growth curve.

Selection on growth components

Selection coefficients, *s*, describe the realized growth advantage of one type over another and are the result in the absence of non-transitive interactions like allelopathy or frequency dependent selection. We can therefore calculate the value of each parameter of the growth curve (keeping the other two constant) for an evolved with a given *s* relative to the ancestral strain as:

$$lag_{evolved} = lag_{ancestor} - \frac{s}{r}$$

$$rmax_{evolved} = rmax_{ancestor}(1 + s)$$

$$K_{evolved} = K_{ancestor}e^{s}$$

I assume *s* = 0.2, which is on the higher end but not out of the range of what is typically observed during the early stages of adaptation in many ALE experiments. The parameters for the ancestral lineage shown in figure 1 are: *K* = 1×10^8 individuals, *rmax* = 0.7, and *lag*= 10 hours. Figure 1 shows the growth curves and area-under-the-curve (AUC) for each evolved strain based on these calculations. The figures were drawn in base R using RStudio version 2026.01.0+392 and the AUC calculated using the DescTools package. Code is available upon request.
